# Imaging and Markers as Novel Diagnostic Tools in Detecting Insignificant Prostate Cancer: A Critical Overview

**DOI:** 10.1155/2014/243080

**Published:** 2014-07-15

**Authors:** Sergey Reva, Alexander Nosov, Roman Novikov, Sergey Petrov

**Affiliations:** Department of Oncourology, N.N. Petrov Research Institute of Oncology, 68 Leningradskaya Street, Pesochny, Saint Petersburg 197758, Russia

## Abstract

Recent therapeutic advances for managing low-risk prostate cancer include the active surveillance and focal treatment. However, locating a tumor and detecting its volume by adequate sampling is still problematic. Development of predictive biomarkers guiding individual therapeutic choices remains an ongoing challenge. At the same time, prostate cancer magnetic resonance imaging is gaining increasing importance for prostate diagnostics. The high morphological resolution of T2-weighted imaging and functional MRI methods may increase the specificity and sensitivity of diagnostics. Also, recent studies founded an ability of novel biomarkers to identify clinically insignificant prostate cancer, risk of progression, and association with poor differentiation and, therefore, with clinical significance. Probably, the above mentioned methods would improve tumor characterization in terms of its volume, aggressiveness, and focality. In this review, we attempted to evaluate the applications of novel imaging techniques and biomarkers in assessing the significance of the prostate cancer.

## 1. Introduction

Prostate cancer (PCa) is the most common male malignancy in the Western world and is the second leading cause of death from cancer in developed countries [1]. In view of huge amount of newly diagnosed prostate cancers every year, the concept of “not life threatening” PCa was developed a couple of decades ago [2]. Currently, the majority of localized PCa cases are believed to take an indolent course.

Active surveillance (AS) of patients with low-risk PCa represents an approach that gains vast interest in recent years. One of the most popular models for detection of insignificant prostate cancer was described in widely acknowledged works of Epstein et al. [2] and Bastian et al. [3], who identified the following criteria based on the preoperative biopsy findings: no Gleason pattern 4 or 5, two or less cores with cancer, and less than 50% of any core involved with prostate specific antigen (PSA) density less than 0.15. For better performance, these criteria were further improved by adding percent of positive cores and prostate volume and by waving the initial characteristics [3].

Available results indicate that AS management through follow-ups including serial PSA, biopsy serial PSA, and biopsy (to enable early intervention upon the signs of disease progression) may reduce overdiagnosis and the subsequent overtreatment, avoiding increased mortality among these patients [4]. Nevertheless, all these models were not devoid of limitations, such as the “trade-off” between sensitivity and specificity described by van der Kwast [4], which resulted in missing approximately 30% of patients with insignificant cancer due to attempts of preserving a good level of specificity [5]. Obviously, approximately 30%–40% of men would have an upgraded Gleason score after prostatectomy [6]. The proposed rational decision was to sacrifice sensitivity and to recruit as much patients for active surveillance as possible, bearing in mind that those who have significant cancer will be predominantly censored at follow-up biopsy, or according to PSA dynamics without any significant risks of being late for radical treatment, due to slow progression of prostate cancer [4]. This is indeed a compromise related to imperfection of predictive models, which still needs evaluation.

In other words, to recognize clinically insignificant prostate cancer (CIPCa) is particularly important in preventing overtreatment of low-grade tumors. One of the main disadvantages in the current CIPCa predictions is related to the virtual appearance of this state. There exists an established dogma that some prostate cancers are insignificant. However, the defined CIPCa volume threshold could be highly arguable (despite the fact that it refers to the most reliable criteria of tumor insignificance), since the biology of cancer is not yet fully understood. Initially, Epstein et al. [2] selected the threshold of 0.2 cm^3^ for insignificant tumors but extended it later, up to 0.5 cm^3^, mostly upon the consensus reached within the urological society and the evidence from the important study by Stamey et al. [5]. Anyhow, tumors under the defined threshold seemed to have favorable visual appearance within the prostate and that was the reason for selecting this volume as a proper value. However, locating a tumor and detecting its volume by adequate sampling is still problematic. For example, anterior tumors appear smaller on biopsy and seem undersampled.

Another constraint in identifying CIPCa is related to tumor aggressiveness. Recently, several authors derived nomograms for the prediction of pathologically confirmed IPC with an accuracy of 64–79% [7–9], though less than those in the original Epstein criteria for pathologically confirmed insignificant prostate cancer prediction [9]. Thus, at an individual level, available instruments cannot safely discriminate between aggressive and indolent tumors and the ongoing extensive work aims at identifying novel biomarkers to improve prediction of disease severity [10–12]. Recent studies found an ability of novel biomarkers to identify prostate cancer, risk of mortality, and association with poor differentiation and, therefore, with clinical significance [13].

The third problem is tumor multifocality. In prostate cancer, tumor areas are more often dispersed throughout the entire gland (rather than representing a densely clustered tumor in a single anatomic location) giving rise to multifocal tumors [14]. A fairly consistent morphologic feature of multifocal prostate cancer is the presence of index lesion, a dominant (as measured by tumor volume) focus, and one or more separate secondary tumor foci of smaller volume [15]. Focal therapy is a new developing hypothesis, which implies targeting a larger and more aggressive index lesion only through surveillance of secondary tumor foci [16]. The main problem is to identify this index lesion or large, poorly differentiated and locally advanced unifocal cancer and avoid the unnecessary focal therapy or active surveillance.

In this respect, appreciable results were derived with use of the multifunctional or multiparametric magnetic resonance imaging (MRI) modalities including spectroscopy, dynamic contrast enhancement and diffusion weighting [17]. Their application would improve tumor characterization in terms of its volume, aggressiveness, and focality.

The above mentioned data suggest a probable lack of patterns for insignificant tumor behavior on needle biopsy. In this situation, several diagnostic tools were proposed to verify and suggest the “real” insignificance of prostate cancer.

In this review, we attempted to evaluate the applications of novel imaging techniques and biomarkers in assessing significance of the prostate cancer.

## 2. Evidence Acquisition

### 2.1. Literature Search

A comprehensive electronic literature search was conducted in January 2013 using the Medline database through PubMed as a search engine to identify all publications relating to clinically insignificant prostate cancer and, particularly, such novel diagnostic tools like multiparametric MRI and molecular (mostly genetic/epigenetic) prostate cancer markers. Both experimental and clinical research studies were considered. Only English language articles were included in the review. The search was conducted using a free-text protocol that included the following terms: prostate cancer, multiparametric magnetic resonance, clinically insignificant prostate cancer, genetic and/or epigenetic factors, PCA3, active surveillance, and focal therapy.

### 2.2. Inclusion Criteria

Attention was given to published materials related to imaging, laboratory, and morphological diagnostic steps of prostate cancer with bound to prospective aggressiveness and clinical significance. Reports from meetings were considered, if they were relevant. Significant studies cited in the reference list of the selected papers were evaluated.

## 3. Clinically Insignificant Prostate Cancer Imaging

### 3.1. Measurement of the Tumor Volume: Historical Background

There is a wide range of options for diagnostic imaging [18]. Modern criteria used to identify insignificant prostate cancer in males are quite similar but the proportion of patients to receive active treatment makes up to 1/3 of the total surveyed, under all studies. To improve these results, some new criteria have been suggested recently, among them—the new imaging tools. One of the most determinative CIPCa criteria is the volume of tumor, which can be assessed clinically with imaging tools. Unfortunately, the most reliable tumor measurement can be performed through a careful pathomorphological investigation only and in the classical works, the cut-off value was established only after prostatectomy [2, 5]. The tumor volume threshold below 0.5 mL is established upon the incidentally detected prostate cancer in a radical cystoprostatectomy series [5]. No information on tumor volume was available in these studies, so the biopsy criteria were used to predict the organ-confined disease with negative margins and the negative lymph node status. Moreover, according to Epstein et al. and Bastian et al., the pathologically confirmed insignificant cancer may be correctly predicted in about 3/4 of patients only [3, 19]. It was suggested that measurements of cancer in the biopsy specimen cannot reliably predict the actual volume of cancer, albeit Dietrick et al. found that a core cancer length of 3 mm or more on 2 and less needle biopsy specimens reliably predicts the presence of significant cancer (0.5 mL or large) [20]. 27% of patients with PCa have a fallacy of cancer's insignificancy. Recent data show inadequate sampling of a tumor using traditional ultrasound-guided biopsy, which leads to an inaccurate grading and estimation of tumor volume in 30–50% of men [21, 22]. The tumor volume was not discussed in this overview for the reason that no relationships were defined between the tumor volume and the clinical outcome in patients undergoing RP at Johns Hopkins Hospital [5]. This situation is being changed in the modern era and now the prostate tumors can be revealed even at very low volumes [23]. Imaging becomes important in the assessment of prostate cancer and the identification of a treatment modality but its value is often controversial. In general, despite some promising results of staging imaging, the amount of the examined patients decreased in the last period, by 63% and 11.4% in low- and high-risk patients, respectively [24]. These changes reflect the more careful use of imaging in the PSA screening era but, obviously, both low- and high-risk patients are sometimes referred for treatment (active surveillance) without the appropriate imaging evaluation [24].

One of the pioneer studies related to preoperative determining of the cancer volume in the prostate gland was performed in 1995 by Cupp et al. Imaging was not used in this research and it is not surprising that identification of tumor volume was unclear, Gleason score could not predict the quantity of cancer and no adequate information was provided for differentiating between clinically insignificant and life threatening prostate cancer [25].

Active surveillance and focal therapy are advocated as potential management options for some patients. However, these approaches face several challenges. One of them is a subpar quality of tumor imaging. It can lead to difficulties in selecting suitable low-risk patients for these options. To overcome these challenges, Turkbey et al. suggested a more extensive application of novel approaches to the assessment of tumors' volume [26]. We reviewed the most useful tools for evaluating the potential insignificance of prostate cancer.

### 3.2. Ultrasound Investigation

Transrectal ultrasound (TRUS) was used for local staging of prostate cancer in some of the past studies but is generally considered insufficient to delineate cancer foci because its sensitivity and specificity vary at around 40–50% [27, 28]. In the early study by Ravizzini et al., in more than 300 men with T1c cancer, the total amount of insignificant PCa preoperatively was 16% and the absence of lesions on ultrasound was not predictive of an insignificant tumor [29]. In this study, of 138 ultrasound reports (58%) available for review from 240 men who underwent radical prostatectomy, almost all (131 case) clearly documented the presence (44%) or absence (56%) of suspicious lesions and the location of ultrasound abnormalities. Lesions on transrectal ultrasound, as well as biopsy results (3 mm or more of cancer, or a Gleason pattern of 4 or 5), were predictive of a significant tumor with a positive predictive value of 83%. The authors concluded that despite the lack of relationship between lesions on ultrasound and pathomorphologically confirmed insignificant PCa, the existence of such lesions was a sign of cancer significance but the absence of lesions was not predictive of an insignificant tumor [29]. Integration of power Doppler into the ultrasound study slightly improved the identification of hypovascular cancer foci in the prostate gland but not the staging accuracy [30]. A relatively new TRUS modification is the contrast-enhanced ultrasound with possible improvement of the prostate cancer foci detection [31] with large microbubbles which increase sensitivity with real-time US. They also can be coated with surface ligands and be more targeted at tumor neovascularity in this case [32]. However, no studies were published on the application of microbubbles in local staging disease, except the pivotal study.

Other prospective methods to detect foci of prostate cancer represent technically improved classical ultrasound modalities (2D/3D-greyscale, Doppler) and new modalities (elastography/MRI/ultrasound fusion). The paper by Braeckman et al. describes a small group of patients who were HistoScanned before scheduled radical prostatectomy. In this study, HistoScanning detected accurately cancer foci of > or = 0.5 mL (i.e., the level of significance volume). The sensitivity, specificity, positive, and negative predictive values of HistoScanning for the cancer foci detection 0.5 mL and more were 100%, 82%, 80%, and 100%, respectively, [33]. Multiple studies show the elastography to be a good addition to the ultrasound investigations for detection of prostate cancer [34]. Walz et al. found that, for correct cancer identification with use of real-time elastography, the sensitivity and specificity were 73.4 and 79.0%, respectively, and the negative and positive predictive values were 83.4 and 67.4%, respectively. The overall accuracy in correct identification of the tumor lesion was 76.5% [35]. The recently introduced Shear Wave Elastography shows a decreased user dependency and an increased prostate cancer detection rates. A pivotal study by Enlund et al. showed a sensitivity value of 96.2%, a specificity of 96.2%, a PPV of 69.4%, and an NPV of 99.6% for the prostate cancer detection, without the indication of the tumor mass volumes [36]. MRI/ultrasound fusion combines the best of both techniques and the available studies show promising prostate cancer detection rates. Application of the proposed method made it possible to resolve lesions of 0.5 cc with a good diagnostic accuracy [37]. Thus, the above listed methods are available for visualizing prostate cancer tumors in a clinically significant volume (0.5 mL and more) with close accuracy. However, no data are yet available on acceptability of these methods for staging of PCa, as well as their importance for the CIPCa detection.

### 3.3. Computed Tomography

Currently, the role of multidetector computed tomography (CT) in prostate cancer detection and staging is very limited. CT sensitivity is reported to be very low (not more than 30%) in local staging [38]. Thus, pure CT is not used in PCa staging.

The present role of positron emission tomography/computed tomography (PET/CT) in pretreatment diagnostic phase in small prostate cancer foci is still argued and its use is not clinically recommended [39, 40]. In fact, various studies demonstrated that PET/CT with 11C/18F-choline and 11C-acetate does not distinguish prostate cancer from normal tissue and benign prostatic hypertrophy or high-grade intraepithelial neoplasia, with a high incidence of false-positive findings. The values of sensitivity, specificity, and diagnostic accuracy reported by recent study comparing 11C-choline PET/CT and histological analysis are very low—72%, 43%, and 60%, respectively, [41]. High rate of false-negative results may be related to the small dimension and low uptake of neoplastic lesions. Foci of prostate cancer are often too small to be detected by PET/CT, which has spatial resolution of about 5 mm [42].

### 3.4. Magnetic Resonance Imaging and Its Derivatives

Tumor volume estimation by biopsy sampling is prone to errors. Moreover, biopsy is targeted mainly at the peripheral zone, so up to 31% of the prostate cancer nodules can be missed, if only the peripheral zone is focused [43]. It can be the biggest limitation to the tumor volume definition and active surveillance as well [6]. Among the imaging modalities, MRI provides the highest spatial resolution of the internal zone anatomy. The highest-quality MRI results can be obtained through a combined use of endorectal coils and phased array body coils at 3T [27]. T2-weighted (T2W) images provide depiction of the zonal anatomy of the prostate and are used to identify low signal areas; herewith cancers that arise in the peripheral zone are low-signal intensity foci, even in small volume [44]. The limitation of T2W images is due to much lower signal intensity from the central gland. The sensitivity and specificity of the conventional anatomical MRI techniques for local staging vary considerably with the selected technique, the radiologist experience, and the population: 14.4–100% and 67–100%, respectively, [27, 45]. MRI findings benefit mostly from the intermediate- and high-risk groups, while the conventional anatomical MRI techniques are not required in low-risk patients, and functional MRI techniques are needed for the accurate insignificant PCa staging [46].

The multiparametric magnetic resonance investigation (mpMRI) is a useful tool for improving prostate cancer detection [47] and a large body of work was published in this area over many years. It can aid in diagnosis and accurate localization and the volume of tumor [23]; mpMRI provides a noninvasive approach to characterizing the anatomy (T2-weighted MRI), angiogenesis (dynamic contrast-enhanced (DCE) MRI), and cell density (diffusion-weighted MRI) of the prostate parenchyma [48]. The available evidence confirms the mpMRI practicability to PCa diagnostics [49].

#### 3.4.1. Magnetic Resonance Spectroscopic Imaging

Magnetic resonance spectroscopic imaging (MRSI) builds upon the fact that different tissues have their specific level of metabolism and different metabolites have characteristic resonant frequency [50]. Indeed, the ratio of choline to citrate related to the Gleason score, suggesting the value of MRSI through the information about cancer aggressiveness [27, 30]. The improved tumor detection rate was reported in several studies. MRSI can also aid in the estimation of tumor volume [51]. Seitz et al. showed that by adding the MRSI, three-dimensional data are acquired from the prostate, with volume elements ranging from 0.24 mL to 0.34 mL [23]. As demonstrated by the study of Fütterer et al., the overall accuracies of T2-weighted imaging in localizing prostate cancer made 67–69% and the localization accuracy increased up to 69%–71% when small (<0.5 mL) tumors were excluded. The localization accuracies of MRSI in the peripheral zone and the central gland comprised 76%–82% and 75%–83%, respectively, which is more precise as compared with that of T2-weighted imaging. The localization accuracy increased from 79% to 85% (on average) for tumors of 0.5 cm^3^ volume or greater. Reading the dynamic images in conjunction with the T2-weighted images resulted in accuracies of 81%–91% for the localization of tumors with volumes of 0.5 cm^3^ or greater [52]. Assuming that the clinical significance of prostate cancer is related to Gleason score value, Villeirs et al. investigated the predictive MRSI (1.5T MRI) capacity, that is, the presence or absence of cancer with Gleason score ≥7 prostate cancer in 356 male patients with the mean serum PSA = 11.5 ng/mL. The estimated MRSI sensitivity was 92.7% for high-grade tumors and 67.6% for low-grade tumors, with a 7.4% false-positive rate [53]. In 158 patients with T1c clinical stage, Zhang et al. found that combined MRI/MRSI (at 1.5T) play an important role in preoperative predicting the pathologic stage of prostate cancer with 80% overall accuracy of staging. Interestingly and more important for insignificant PCa detection, the highest accuracy was observed in tumors of <0.5 mL volume (91%), with the staging accuracy of 75% for tumors >2.0 mL [54]. In contrast, the American College of Radiology Imaging Network in a prospective multicentre study found that the accuracy of combined MRI/MRSI was equivalent to that of MRI alone [55]. It is noteworthy that from all man with suspicious for prostate cancer foci on the mpMRI, only 6% had a lesion on MRSI alone and 40% on DCE plus MRI [56, 57].

#### 3.4.2. Magnet Strengths and Prostate Cancer Detection

With reference to magnet strengths and the rate of prostate cancer detection, Labanaris et al. reported a comparable lesions prevalence (65%) between different groups (3T magnet and 1.5T magnet) using T2 sequences on a 1T magnet only [58]. Other authors obtained detection rate of 45% in groups using a 1.5T magnet without advantage of higher magnet strength [59–61]. Similarly, Vargas et al., assuming the MRI results in prostate cancer detection rate, found no differences between 1.5T and 3.0T studies [62]. Based on these data, we can agree with Park et al. that sequences, such as T2-weighted imaging, remained unaffected from the technological improvements in the last decade which still represents the mainstay of prostate MRI and the theoretical advantages of 3T MRI still have not been validated [63]. However, Durand et al. investigated a feasibility of 7.0 tesla MRI of the human prostate ex vivo in 12 patients after radical prostatectomy. After the specimen's section, the whole gland was imaged on 7.0T MRI system. The authors concluded that the macroanatomy of prostatic tissue was well defined on MRI. This could be helpful in the assessment of the volume and differentiation of prostate tumors. Moreover, the authors produced the first magnetic resonance angiogram of the prostate tissue. This may be helpful to assess the correlation between disruption of normal vascular patterns and the size or grade of tumor and clinical behavior [18]. Unfortunately, nononcological conditions (e.g., prostatitis and postbiopsy changes) may influence the investigation's results [23].

#### 3.4.3. Diffusion-Weighted MRI

Diffusion-weighted MRI (DW-MRI), if applied in addition to conventional T2-weighted and contrast-enhanced MRI, improves tumor detection and localization. Several studies showed an increase of sensitivity and specificity, from 71% to 89% and from 61% to 91%, respectively, when DW-MRI is combined with T2W-MRI, as compared with the respective values 49–88% and 57–84% for either diagnostic modality taken separately [64–66]. Diffusion-weighted imaging (DWI) is the most practical and simple to use [47]; it has shown promise for the assessment of tumor aggressiveness by quantifying random Brownian motion properties of water molecules (diffusion) in tissue and displaying it in the form of the apparent diffusion coefficient (ADC). Taking this into account, DW-MRI could provide great benefits if used to differentiate abnormal tissues at early stages [67]. Initially, most studies concerned with DW-MRI were focused on the peripheral zone of the prostate, due to the prevalence of cancer foci in this area and difficulties in detecting prostate cancer in the transition zone (TZ) of high cellularity [68]. Recent studies showed that DW-MRI performs better in the TZ than T2W-MRI [69, 70]. Moreover, tumor foci localized in the TZ are more likely to be insignificant. Oto et al. compared 38 tumor foci and the same number of nodules of stromal hyperplasia and glandular hyperplasia in the TZ with significant ADC differences between groups [71].

In one study investigating the local staging accuracy of 3-T endorectal MRI, for experienced radiologists, the sensitivity of local staging was 88% and the specificity 96% [72]. Encouraging results were obtained in linking correlating the mpMRI components and the tumor aggressiveness. Both DWI [73] and the creatine-plus-choline-to-citrate ratios in MRSI were associated with Gleason score [74]. In a recent analysis, the ADC value at DWI represented the best performing single parameter for prostate cancer detection, when compared with T2-weighted imaging and DCE-MRI parameters [75]. Thereby, assessing tumor aggressiveness by these techniques can aid in evaluating risk parameters and selecting candidates for active surveillance. In a similar study by Hambrock et al., ADCs at 3.0T showed a relationship to Gleason grades in prostate cancer with a high value in the differentiation of low-, intermediate-, and high-grade cancer [73]. In the analysis of 70 patients, 56 had clinically significant tumors (>0.5 mL) and tumors in the peripheral zone only were noted. Although the low ADCs in most tumors were attributed to the increased cellular density, these results could also be influenced by fibrosis, type of organization, and shape [74]. Furthermore, despite the fact that lower ADC values seem to be associated with higher Gleason score (due to the higher cellular density in tumors with poor differentiation), an overlap between the ADC values and different Gleason scores could be observed [75]. Such circumstances can theoretically reduce the DW-MRI benefits. However, in 94% of tumors, ADC was in exact concordance with the most aggressive tumor composition despite the fact that the DW-MRI value was less in few cases of severe motion artifacts, widespread intraprostatic hemorrhage, or ghosting artifacts on the MR images. In this study, none of the patients had tumor volume less than 0.5 mL and a Gleason grade more than 3 [75]. With regard to the CIPCa, DW-MRI enabled better treatment selection (as an mpMRI component), provided a more representative Gleason score, and enabled better risk stratification and follow-up for patients treated with active surveillance protocols. Possibly, the DW-MR imaging application can include differentiating patients in need of active treatment [31] and the early noninvasive identification of such patients could be helpful in avoiding the unnecessary surgery [69]. However, in the more recent study of this group, we can see a broad insignificant PC definition—different for surgery and radiotherapy. For radical prostatectomy, tumor volume >0.5 mL was applied, as well as Gleason grade > 4 and nonlocalized cancer (stage > T3a/N1), whereas, for men who chose radiotherapy or active surveillance, tumor volume had no significance and the definition incorporating Gleason > 4, PSA > 10 ng/mL, or PSA density > 0.15 was used [74]. In both cases, 93% of cancers diagnosed using DW-MRI were clinically significant on image-guided biopsy.

#### 3.4.4. Dynamic Contrast-Enhanced (DCE) MRI

DCE-MRI with the low molecular weight contrast agent enables noninvasive imaging of tumor angiogenesis. Prostate cancer demonstrates angiogenesis that could be detected by DCE-MRI. Early rapid enhancement and early washout on DCE-MRI images is highly predictive to prostate cancer but not pathognomonic. Smaller and low-grade tumors may not be visualized on DCE-MRI; this finding is highly important in the era of overscreening of the prostate cancer [50, 76]. In a study of 83 patients, Puech et al. assessed the prostate cancer volume at histopathology with the performance of 1.5T DCE-MRI for identifying and localizing tumor foci. The sensitivity of identification of prostate cancer foci of any volume was low (32%), with the specificity of 95%. However, the sensitivity and specificity for identification of cancer foci >0.5 mL were 86% and 94%, respectively, [77]. Seitz et al. compared two functional methods (DCE and MRSI) in their previously mentioned review and noted that sensitivity, specificity, and accuracy in tumor staging (without indications on insignificant PCa) were higher with DCE-MRI than with MRSI [23]. The overall sensitivity, specificity, PPV, NPV, and accuracy for predicting prostate cancer detection with combination of MRSI plus DCE-MRI are quite high—93%, 89%, 89%, 93%, and 91%, respectively, in study by Sciarra et al. Notably, only 3% of patients in this group had lesions on DCE alone and 40% on DCE plus MRS [57]. Even so, to predict insignificant PCa, we need the more comprehensive information (localization, volume, and aggressiveness of the tumor).

#### 3.4.5. Multiparametric MRI (mpMRI): Limitations and Its Effect on CIPCa Diagnosis

According to the major cited studies, MRI techniques can provide the needed information about the location and size of prostate cancer (particularly, in case of its clinical insignificance), as well as its relationship to prostatic structures, which may be valuable both in active surveillance and focal treatment [47]. An important implication to mpMRI is an attempt to avoid the overdiagnosis of prostate cancer. Recent studies showed that Gleason score was 6 and less for 90% of cases with nonsuspicious MRI, normal DRE, and PSA level 3–10 ng/mL [59]. Subsequent biopsies corresponded to that of insignificant tumors in 88% of cases. Another study of biopsy-naive men revealed that more than one-third (38%) of patients passed a MRI without suspicion for disease and a quarter of these (23%) with a clinically significant cancer (cancer core length less than 5 mm and/or any Gleason pattern not more than 3) only in 2.3% cases [60]. Finally, early results of the PROstate MRI Imaging Study (PROMIS) were presented by Ahmed et al. at the annual EAU congress (Paris, 2012). This ongoing study would provide an answer, whether mpMRI can discriminate between men with no cancer or clinically insignificant cancer versus those with clinically significant cancer. According to the initial data, PROMIS is powered to measure an increase in sensitivity from 48% in TRUS biopsy cases to approximately 70% in mpMRI cases. PROMIS will provide level 1 evidence for the diagnostic utility of using mpMRI and in the future would allow to safely avoid a biopsy annually in patients on active surveillance and/or improve the detection of clinically significant prostate cancer by one-fifth [48, 78]. Thus, mpMRI may play a role in guiding the patient selection for active surveillance, as well as potentially monitoring tumor volume noninvasively [21].

However, several related problems still persist in setting the mpMRI diagnostic role. One of the most complicated is variations in protocols and the lack of robust diagnostic criteria. The absence of systems to quantify the risk of significant prostate cancer poses another challenge. To overcome these problems, the European Society of Urogenital Radiology (ESUR) has recently published a scoring system for mpMRI—Magnetic Resonance Prostate Imaging Reporting and Data System (MR PI-RADS) [78]. According to this system, the mpMRI features are characterized as level of suspicion for each MRI sequence, from 1 to 5, the sum of the ESUR scores (range: 3–15).

The proposed ESUR scoring system has been further validated in a real-life study by Portalez et al. [79, 80]. When compared with biopsy results, the authors found that the ESUR scores showed higher level with positive cores. The presence of cancer increased proportionally to T2W and DWI ESUR scores, whereas an indentation was observed for DCE MRI. The ESUR-S showed an excellent, clinically relevant predictive characteristic and threshold value was 9. The ESUR-S showed a higher positive predictive value (58.0%) and accuracy (89.1%). This question will be more closely reviewed in the ensuing schedules.

Still, because of suboptimal sensitivity, some cancer foci would be missed. In the present cohort, 1286 cores were taken in ESUR-S <9 areas, of which 64 cores (4.9%) were found positive for cancer. Conversely, restricting the biopsies to ESUR-S ≥9 would have detected 136 of 200 positive cores (68%). Hadaschik et al. reported a 96% detection rate for any cancer in case of “highly suspicious" mpMRI-lesions, 71% detection rate for “any suspicious” lesions, and 35% of men with prostate cancer diagnosed by MRIs as “nonsuspicious” [81]. This study showed the highest PPV in “strongly suspicious” MRIs (96%), whereas the same changes were associated with PPV of 91% by Sciarra et al. [56]. Similar work by Pinto et al. supported these findings and showed that stronger MRI signal increased the likelihood of prostate cancer detection with PPV of 90%, 67%, and 30% for MRIs investigation with strong, moderate, and low suspicion, respectively [82].

Although this cognitive process was shown to achieve the relevant accuracy in mpMRI-suspicious foci, the process remained prone to imprecision, as evidenced by the differences in detection rates among operators of similar experience [59]. Different expedients have been proposed. One is the use of elastic surface registration to overcome these limitations. This technology was shown to prove 100% accuracy and millimetric precision randomly located 0.5 mL hypoechoic lesions [83].

Also, the important factor is the radiologists' experience. Experienced radiologists who are trained appropriately to read it are still lacking. This question was highlighted in a recent study by Vargas et al. In this study, three radiologists interpreted independently and assigned each one a score indicating that tumor tissue was definitely evident, definitely absent, probably absent, or indeterminate. Those radiologists, who had read less than 50 prostate MRI scans at the time of this study had consistantly less accurate readings than those, who had a fellowship in body imaging plus specialized training in prostate imaging plus had interpreted approximately 500 MRIs, and than radiologists, who had completed a fellowship in genitourinary radiology and had read more than 5,000 MRI scans [62]. This data confirm the importance of training and experience for the accurate interpretation of MRI scans of the prostate.

In relation to the active surveillance based on the mpMRI data risk stratification, patient and family anxiety is also a potential barrier to accept it. Indeed, some can find the idea of doing nothing against their disease inconceivable. To overcome this problem, an adequate patient education should be provided for reassurance about the risk of such cancer getting worry and the chances to detect these changes by mpMRI techniques in time.

Another problem is the cost of investigation. This is crucial to determining whether this is affordable for men presenting for prostate cancer risk assessment [84]. The previously mentioned study PROMIS would evaluate the MRI benefits as the first tool to be applied to men with an elevated PSA level or an abnormal DRE [78]. Another study, Smart Target, sets the goal to translate lesions suspected on the MRI to an ultrasound-based platform, which can reduce the cost of investigation. These articles could give us answers about the utility and cost benefits of mpMRI and MRI-guide biopsy in the prostate cancer detection [84]. In a prospective study by Riches et al., the combination of DWI with either MRSI or DCE-MRI increased the area under curve (AUC) values for detection of prostate cancer tumors 1 mL and more from 0.65–0.71 to 0.94; adding a third functional MRI technique did not further improve detection (the AUC value for all functional MRI techniques was 0.95) [85]. On the other hand, the addition of DWI and DCE-MRI to T2-weighted MRI increased the accuracy in detection of transition-zone cancer from 64% to 79% [86].The cost effectiveness of mpMRI should be assessed against achieving a more accurate initial insignificant PCa diagnosis and the feasibility of tailored therapies with lower costs and more effective active surveillance.

In addition, the use of mpMRI sequences, such as DW-MRI and DCE-MRI, is clinically available for only a few years. To the best of our knowledge, the most exhaustive published study on the mpMRI assessment of patients suitable for active surveillance includes only 60 male patients [87].

Previous literature reviews indicated an increasing mpMRI role combining T2W imaging, DWI, DCE, and MRSI as the most effective available instruments for imaging the prostate cancer, particularly, clinically insignificant or low-risk forms [47]. There is no doubt that to ideal PC diagnosis spatial resolution in MRI continues to improve, but already it is not only an aid in diagnosing prostate cancer but also a source of information that complements routine histological findings, thereby providing clinicians and patients with a more complete understanding of the disease [18].

## 4. Markers in the Diagnosis of Prostate Cancer

### 4.1. Prostate Specific Antigen (PSA): PSA Derivatives and Their Contemporary Role in CIPCa Diagnosis

#### 4.1.1. Prostate Specific Antigen

The most accurate and applicable marker for prostate cancer diagnosis since the mid-1980s is prostate specific antigen (PSA) [88]. Despite the commonly known fact that the serum PSA test does not represent the ideal tumor marker, it is still the most frequently used in the diagnosis and management of PC and the available data indicate that the PSA-based screening can reduce disease-specific mortality [89]. The European Randomized Study of Screening for Prostate Cancer (ERSPC) trial reported that PSA screening was associated with a 20% relative reduction in prostate cancer mortality at a median follow-up of 9 years, resulting in reduction of about 7 prostate cancer deaths per 10,000 men screened [90]. However, the mortality benefit came at a cost of overdiagnosis, as the incidence of prostate cancer in the screened population was significantly higher than the incidence of men presenting the clinically significant disease. In other words, PSA screening was associated with a large amount—approximately 76%—of false-positive results [91].

On the other hand, there is a strong association between increased PSA levels and the increased risk of PCa mortality. In a recent study, Ørsted et al. showed, that even at PSA levels below common thresholds for biopsy, 3.0 or 4.0 ng/mL, the development of surveillance programs for men with lower PSA levels may be justified [92]. Results of this study based on prospective evaluation of 4383 men aged 20–94 years from the Danish general population support the idea that the findings on PCa incidence based on PSA screening are clinically relevant and not driven by detection of insignificant PCa.

#### 4.1.2. PSA Density

Although the rationale for AS is well established, there is no consensus regarding the optimal characteristics of patients who should be managed by this strategy [93]. Selection is usually based on prostate biopsy results and the values of PSA and/or its derivatives, such as PSA density.

The importance of PSA and its derivatives (PSA density, PSAD) in the CIPCa detection was first indicated in the classical study undertaken by Epstein et al. in 1994 [2]. In this work, the best model predicting insignificant tumor was PSA density less than 0.1 ng/mL per gram, with no adverse pathologic findings on needle biopsy or PSA density of 0.1 to 0.15 ng/mL per gram, with a low- to intermediate-grade cancer smaller than 3 mm found in only one needle biopsy core specimen. The authors concluded that serum PSA level and PSA density, as well as biopsy pathologic findings, are accurate predictors of tumor extent [2]. Since then, we have no exact cut-off value of the PSA density eligible to insignificant PCa and its threshold varies in different studies ranging between 0.1 and 0.2 ng/mL [94, 95]. Moreover, Epstein's pathologic definitions are quite clear for “insignificant” and “minimal” prostate cancer (tumor volume less than 0.2 mL and 0.2–0.49 mL, resp.) but not for PSA and PSAD. Also, no PSAD differences were noted between the insignificant and the minimal prostate cancers, with the maximum level of 0.25 ng/mL per gram in both cases [2]. Nevertheless, predictors of the overall tumor category based on either PSA level or PSA density were highly statistically significant predictors of the final tumor category. PSA level, PSA density greater than 0.1 ng/mL per gram, and PSA density greater than 0.15 ng/mL per gram were associated with positive predictive values of 88%, 91%, and 95%, respectively. However, these tests showed poor results for cases of insignificant tumors, with negative predictive values of 43%, 42%, and 33%, respectively [2]. Several authors generated preoperative predictive models to better identify potential CIPCa and most of these monograms' included PSA [7, 9] or PSA density [96]. These monograms' are slightly superior to the original Epstein criteria in their ability to predict CIPCa [97].

In recent study, in all patients eligible for active surveillance, PSA was >10 ng/mL in 10% of the surveyed patients and PSA density was >0.15 ng/mL per milliliter in 32% [98]. Contrary, Sengupta et al. showed that only those patients with PSA less than 10 ng/mL were considered as really “insignificant” [99]. However, the low PSA level does not necessarily mean a “clinical insignificance.” In the study of Walz et al., the incidence rates of prostate cancer in men with PSA < or = 2.5 ng/mL were 16.5% and 36.5%, if these patients had a pT3 stage and Gleason score, respectively, [100].

A recently proposed updated histopathologic definition of CIPCa does not include PSA again [101]. Previous studies reported that men with PSA density less than 0.15 ng/mL per gram, along with less than 3 positive biopsy cores, 50% or less of any core involved, and a Gleason score 6 or lower are likely to have CIPCa in their radical prostatectomy specimen [102].

#### 4.1.3. PSA Kinetics (Velocity and Doubling Time)

Another PSA derivative of feasible importance is PSA velocity (PSAV), though the available data are also quite controversial. A few retrospective studies supported the value of this parameter. The importance of PSA velocity was first suggested by Sengupta et al. who reported in 2008 that the PSA doubling time (PSA DT) was a CIPCa predictor, but only in a multivariate model with accepted pathological criteria [99]. This finding was supported by Loeb et al. In their study, PSA velocity >0.4 ng/mL per year was associated with twofold decrease in the probability of insignificant cancer (10% versus 5%) [103]. The landmark study by D'Amico et al. demonstrated that a rapid rise of the pretreatment PSA level was associated with prostate cancer significance [104]. Carter et al. showed that PSA velocity 15 years prior to diagnosis was higher in patients who died from prostate cancer [105]. In most studies, high PSAV/shot PSA DT was an indication for active treatment but the threshold is different between simply “rising PSA” [106, 107], PSAV >1 ng/mL per year, and PSA DT <2-3 years [108, 109]. To improve the results of active surveillance, the PSA DT was increased up to 3 years in a more recent study of this group [110]. PSA DT made the second reason for active treatment (in 13–48% cases) preceded by the histological reclassification (27–100%), according to the systematic review by Dall'Era et al. [111]. The authors referred to study by Krakowsky et al., where all 5 patients who died from PCa had had a rapid PSA DT of less than 1.6 years [112]. Also, PSA DT represented one of two parameters associated with high-grade or higher volume at repeat biopsy within the PRIAS cohort of men eligible for surveillance after the first biopsy [113]. Thus, in general, PSA kinetics plays an important role for predicting disease progression in active surveillance. Interesting data from the Royal Marsden cohort supported the importance of PSA changes and showed that PSAV (more than 2.0 ng/mL per year) could be more predictive than PSA DT and closely associated with Gleason grade score from 6 to 7 and more in >50% of the cores positive for cancer [114]. However, other prospective studies did not identify PSA velocity as a CIPCa predictor [115, 116] and even provided some discrepancies in PSA DT definition; for example, Toronto group used at least eight PSA values to assess risk. After a median follow-up of 6.1 years, 38% had at least one PSA value >10, 37% had a PSA DT <2 years at least once, and 42% had a PSA velocity >2 ng/mL per year at some point [117]. Johns Hopkins group showed no correlation between PSA DT and adverse pathology at surveillance biopsy or surgical treatment [118]. Currently, PSA velocity is considered an inappropriate indicator of CIPCa and the use of this parameter led to appreciable numbers of missed indolent but also significant cancers in the active surveillance program [118]. In certain cases, the information about the short doubling time comes late [119].

Thus, the refined PSA testing and PSA kinetics (i.e., PSA DT or PSAV) are not good predictors for CIPCa as such but they play an important role in identification and follow-up of the screened cohort. The accuracy of prostate biopsy, PSA, and its derivatives in evaluating pretreatment prostate cancer risk and prognosis is known to be limited [98, 120].

### 4.2. Prostate Cancer Antigen 3

Currently, there is much evidence that novel markers play an important role in the prostate cancer diagnosis. One of the most discussed is the prostate cancer antigen 3 (PCA3) described by Bussemakers in 1999 and considered as possibly useful by de Kok et al. in 2002 [121, 122]. Several studies investigated the role of prostate cancer antigen 3 (PCA3) in the insignificant cancer diagnosis and some of them demonstrated a significant correlation between PCA3 score and tumor volume [123, 124], extraprostatic extension [125], and Gleason score [8]. The available data related to the CIPCa are quite contradictory. Auprich et al. confirmed that PCA3 score could serve as a CIPCa indicator. Including the PCA3 score into multivariable models increased the accuracy of CIPCa prediction [118]. Also, more recent studies confirm this result with high diagnostic accuracy (equal to 86.8%) of PCA3 score cut-off >20 in the presence of a microfocus of significant prostate cancer [126]. Contrary to these data, Durand et al. suggested that the PCA3 score of >35 represented an independent predictor in a multivariate analysis of a tumor volume >0.5 mL [127]. All these authors concluded that PCA3 scores correlated to numerous histoprognostic factors (specifically, the tumor volume) and these results could affect clinical selection of patients eligible to undergo active surveillance in the near future. However, no correlation was found between PCA3 and the clinical and pathological stage in the study of Hessels et al. [128]. Accordingly, Deras et al. found that PCA3 testing was independent of tumor volume and thus questioned the value of this method for active surveillance [129]. According to Nakanishi et al., the total tumor volume and prostatectomy Gleason score were significantly correlated with PCA3 score (but not with other clinical and pathological features) and the latter was significantly different when comparing low volume/low-grade cancer (dominant tumor volume less than 0.5 cc, Gleason score 6) and significant cancer. Moreover, PCA3 made the best predictor of total tumor volume in prostatectomy and could discriminate low volume cancer (total tumor volume less than 0.5 cc), which could be important in the view of the currently observed association of PSA and cancer volume. The authors found that PCA3 threshold of 25 was good to discriminate between total tumor volumes less than 0.5 cc and 0.5 or greater cc. At this cut-off the observed diagnostic accuracy was 76%, with a high odds ratio of 7.3 to predict total tumor volume less than 0.5 cc. 94% of patients with a significant prostate cancer on the prostatectomy specimen (the dominant tumor volume 0.5 cc or greater and/or Gleason score 7 or greater) had a PCA3 score of 25 [8]. Based on these data, Marks concluded that PCA3 testing could become an important tool to help us decide who should undergo treatment [8]. However, while association between PCA3 level and tumor size and aggressiveness is encouraging, it remains to be proven that patients with low PCA3 levels can be safely observed [8]. Another study from Haese et al. shows that PCA3 score is associated not only with the clinical stage, Gleason score, but also with clinically significant versus indolent prostate cancer (cT1c, PSAD < 0.15 ng/mL, Gleason score 6 and less, positive cores <33%). In this study, men with cT2 had a higher PCA3 score than those with cT1c and, similarly, men with Gleason score 7 and more had a higher PCA 3 score than those with Gleason score 6 and less. All the men with significant prostate cancer (72 patients, 83%) had a PCA3 score higher than patients with CIPCa. The maximum specificity was reached in a group with PSA level of 4–10 ng/mL (“grey” zone) [130]. Similar study showed that PCA3 cut-off of 35 provided an optimal balance between sensitivity and specificity. However, many urologists urge to avoid the risk of missing significant cancers and use a PCA3 score cut-off of 20 that would reduce the number of biopsies by 44%, while missing only up to 10% of cancers with Gleason score 7 and more [131]. This threshold may have the highest utility regarding the decision of whether the cancer found on repeat biopsy is clinically significant. Tosoian et al. assessed the value of PCA3 within an active surveillance program. In 294 men, the annual surveillance biopsy results were evaluated and matched against the PCA3 score. Patients with progression on biopsy had a mean PCA3 score similar to that of men without progression [132]. In general, we can conclude that the PCA3 test is not capable of replacing the PSA and its derivatives as a tool to assess the risk of prostate cancer significance in clinical practice [133]. Data relating to the accuracy of prostate cancer staging are contradictory and PCA3 as a prognostic test should be subjected to further studies. Furthermore, we do not know about the reliable cut-off PCA3 value between types of prostate cancer with different biological behavior. Based on the data reviewed, Turkbey et al. also suggested that novel biomarkers could help with risk stratification but they are not yet sufficiently validated to replace classical parameters (Gleason grade, tumor volume, and tumor stage) [26].

### 4.3. Immunohistochemical Markers

Certain immunohistochemical findings (markers against basal cells (high-molecular cytokeratin, p63), PSA, and alpha-methyl-coenzyme A racemase) could be useful for the diagnosis of some potential CIPCa. However, nonspecific staining of prostate cancer cells with high molecular weight cytokeratin (HMWCK) can lead to false-negative diagnoses. P63 represents a more specific marker for basal cells than HMWCK [134]. Despite the fact that both are useful in prostate cancer diagnosis, all these tests can provide considerable false-positive and false-negative results [102]. At present, we have no trial results with high level of evidence concerning the relationships between insignificant PCa and these novel markers.

### 4.4. Novel Cancer Markers

The new strategy is to find significant changes in genetic and epigenetic factors related to the prostate cancer persistence. Recent register-based family studies supported the existence of a genetic component in the prognosis of various cancer types including prostate cancer [135]. These data suggest that genetic markers could identify men at risk for progressive disease.

#### 4.4.1. Genetic Markers and Their Associations

Trock et al. investigated novel genetic markers, adenomatous polyposis coli (APC), and glutathione-S-transferase P1 (GSTP1), with the aim to assess a risk of cancer in the biopsy specimens. This study involved patients with high index of suspicion of prostate cancer; APC and GSTP1 methylation ratios below the threshold (predicting no cancer) produced an NPV of 96% and 80%, respectively, indicating that APC has significantly higher NPV. However, combining these methylation markers produced a result similar to that of APC alone [136]. The factor of importance was difficult to assess, due to the fact that GSTP1 specificity was higher than that of APC and suggesting that methods would be more beneficial when both markers are used. The authors concluded that including these DNA methylation markers into the protocol of patient examination prior to repeat biopsy could reduce the unnecessary investigations posterior to the initially negative biopsies. Another study evaluated methylation markers in previously negative biopsies found that sensitivity for detection of cancer on subsequent biopsies was 52–84% [137]. All the above mentioned studies considered the presence of prostate cancer only and disregarded tumor volume or Gleason scores. Thus, data of these works cannot be linked with potential CIPCa.

Since prostate cancer has a strong heritable component and the family history should be considered as a risk factor for developing the disease [128], an unbiased genome-wide search for inherited variants of prostate cancer associated risks is reasonable. This approach is called a genome-wide association study (GWAS); it implies scanning the entire genome and evaluating common inherited variants in large number of cases [138]. Currently, several prostate cancer single nucleotide polymorphisms (SNPs) have been identified as associated with prostate cancer development through GWAS with attention to the ability to distinguish between cancer aggressiveness [139, 140]. The results are yet inconclusive; some data demonstrate the relationship between risk variants and the more aggressive disease, while no associations were revealed in the others [141]. The transmembrane protease, serine 2 v-etserythroblastosis virus E26 oncogene homolog (TMPRSS2-ERG) translocation presented in many prostate cancers [142]. Detection of TMPRSS2-ERG fusion in urine was reported to yield >90% specificity and 94% positive predictive value in prostate cancer detection. Demichelis et al. demonstrated that PCa containing the TMPRSS2-ERG expression gene took a more aggressive course, possibly mediated through an increased ERG gene expression [143]. However, a clinical diagnostic test and relationship with CIPCa are yet unavailable [144, 145]. This issue remains quite controversial for evaluation of the PCa prognosis. We can see an overlap between most of the studies and genetic markers, and, as a result, the difficulties in clinical implementation are obvious. Another question is the reproducibility of a genotype of small tumors in case of consistent changes in a way [146]. Several studies suggested risk variants to predict disease progression among patients with clinically localized prostate cancer; however, findings between these studies are not consistent and the data are limited in sample size [147, 148].

Another novel biomarker is beta-microseminoprotein (MSMB); its expression is reduced by the rs10993994 risk SNP in the MSMB promoter. The MSMB levels are low in prostate cancer, as compared to the benign prostate disease. Decreased urinary levels were shown to improve upon urinary PSA, but not serum PSA, for prostate cancer diagnosis [149].

There is some evidence that prostate cancer aggressiveness has a heritable component [135]. One allele associated with risk of developing prostate cancer is also believed to be associated with aggressive prostate cancer [150]. In the study by Gudmundsson et al., KLK3 encodes PSA was one of the six loci identified by GWAS that were associated with higher PSA levels in patients without prostate cancer [151]. Additionally, novel prostate cancer risk variants at the chromosomal locus 8q24 were identified several years ago by these authors and under the genome-wide association study by Yeager et al. [152]. The majority of available studies on genetic variants that could affect the aggressiveness and prognosis of prostate cancer were focused on alleles associated with prostate cancer risks. Perhaps, it will be important to perform true GWAS due to heterogeneity in defining disease aggressiveness and outcome because different clinicopathologic criteria have been used to define less aggressive disease across sites even with the same study [134].

Additional important information related to the prostate cancer significance form GWAS is that 33 risk-associated SNPs have been identified [153, 154]. These SNPs are associated with prostate cancer risk [137]. Particularly, the genetic score based on a combination of these risk-assessed SNPs can predict a risk of prostate cancer in a certain individual [155, 156]. In one of these studies, Duggan et al. identified seven SNPs (from a huge amount of approximately 60,000) that had an association with the risk of aggressive prostate cancer. Additional analysis of distribution of these SNPs indicated that one SNP, rs1571801, was associated with the aggressive prostate cancer. However, no CIPCa patients (by the standard criteria) were involved in this study despite the large total number (1242 men) [153]. In the newest analysis of the REDUCE trial, men had prostate cancer risk based on clinical parameters only: 23% for any prostate cancer and 6% for high-grade prostate cancer; after adding the genetic score, the prostate cancer detection rate was 14% for any prostate cancer and 4% for high-grade prostate cancer [155]. However, the authors observed that the benefit of adding the genetic score (based on 33 established prostate cancer risk-associated SNPs) was the greatest among men at intermediate risk and for high-grade (Gleason score 7 and more) prostate cancer. Moreover, in the prostate cancer low-risk group (potentially insignificant), the genetic score may provide a limited clinical impact [157]. Multiple studies demonstrate that SNPs do not differentiate between indolent/insignificant and aggressive prostate cancer [156, 157]. Another recent attempt to estimate the probability of cancer progression in the PROCAR (Progression in Cancer of the Prostate) study with 23 established prostate cancer susceptibility variants (SNPs) in a large cohort of men was also unsuccessful. In this study, patients experiencing disease progression were more likely to be on active surveillance [158].

However, certain genetic alterations were correlated with the prostate cancer significance [159, 160]. The impact of other known genetic alterations on clinical behavior or tumors remains unclear and somewhat controversial; for example, TMPRSS2-ERG was involved as both negative and positive prognostic marker [161]. Another SNPs gene, DAB21P, despite expressing in normal prostate tissue, has a decreased expression in prostate cancer cells [162]. Besides, that could be more important, the reduced expression of DAB21P correlated with the increased expression of the transcriptional repressor EZH2, which proved to be one of the strongest markers of aggressive prostate cancer [163]. Thus, the proteins encoded by this gene are attractive candidates for a prostate cancer aggressiveness risk gene [153].

Another attractive marker for understanding the behavior of a particular cancer is gene expression. There exist multiple challenges in the interpretation and standardization of assessing gene expression. One of the problems is that the prostate cancer itself can be highly heterogeneous and multifocal. Different Gleason grades identified within an individual prostate specimen suggested this opinion [164]. Penney et al. found the differences in gene expression between and clinical behavior in patients with Gleason 6 and less versus Gleason 7 and more [165]. Other groups found that expression levels of other genes (stem cell-like states, markers of increased cell-cycle progression) would reflect a more aggressive disease, and, thus, can reflect CIPCa [158, 166]. However, up to date there is no consensus regarding which markers can be used for assessing prognosis and, especially, for determining insignificant PCa.

Another important group is epigenetic mechanisms, which are defined as inheritable that alter expression without changing gene. This mechanism was discovered about 25 years ago by Vogelstein who suggested an assessment of GSTP1 hypermethylation as a marker of central part of prostate carcinogenesis [167]. These mechanisms involve modification of DNA, modifications of mechanisms of the DNA-supporting proteins, and expression of noncoding RNAs (ncRNA) and its small form miRNA. The loss of epigenetic control is common in cancer. The data about miRNAs expression in prostate cancer are published since 2007 and caused enormous interest because of up- and downregulation when malignant and benign prostate cells were compared [168]. Many miRNAs are expressed in prostate cancer but this miRNAs often converge on key carcinogenesis pathways and it is difficult sometimes to distinguish those possibly relevant to prostate cancer staging and prognosis [169]. Catto et al. noticed that altered miRNA expression is common and appears important in PCa [170].

Most studies showed the existing correlation between some types of miRNA and the advanced prostate cancer [171, 172], but until now we have no clear strategy on how to use miRNAs in the prostate cancer detection. However, recently, Brase et al. screened the serum for 667 miRNAs in localized cancer patients and found that miR-141 and miR-375 showed significant value as diagnostic and prognostic biomarkers [173]. Moreover, Moltzahn et al. recently defined a new diagnostic miRNA panel with different (previously identified) miRNAs [174]. The fact that miRNA expression is dynamic and will differ with the phases of prostate carcinogenesis, including insignificant or indolent or low-grade, seems to be very promising.

The potential benefits of applying risk models in clinical practice are yet to be demonstrated. Moreover, it is unclear, whether using genetic and epigenetic factors to define an early or intensive screening group would lead to predict the progression in the low-risk group during active surveillance: the former group could still be subject to morbidity from diagnosis and treatment from overscreening for potentially nonlethal prostate cancer. The diagnosis of aggressive cancer may be delayed in the latter group. Kader et al. showed that adding the genetic score represented an up to 25% reduction in unnecessary biopsies [157]. However, more recent studies found an ability which was not only an identified prostate cancer and risk of mortality but also an association with poor differentiation and, therefore, with clinical significance.

## 5. Conclusions

Despite the numerous clinical, laboratory, and morphological criteria are available to date, development of nomograms and drastic selection of patients with low-risk prostate cancer, current criteria for AS, and focal therapy cannot avoid a significant number of patients with upstaged and upgraded disease at diagnosis. Therefore, decision-making in treatment and surveillance monitoring should be improved through a more extensive application of new biomarkers and imaging techniques. The most challenging to date refer to epigenetic factors and multiparametric MRI. These methods can contribute to decision making by choosing a focal therapy as an appropriate treatment. Currently, transrectal ultrasound (TRUS) biopsy protocols appear inadequate for the selection of patients for active surveillance and focal therapy. Obviously, introduction of novel markers and advanced imaging techniques, such as multiparametric magnetic resonance, is required.
